# Incidental Sacral Meningocele in an Elderly Patient Diagnosed After Epidermal Inclusion Cyst Removal: A Case Report

**DOI:** 10.7759/cureus.27385

**Published:** 2022-07-28

**Authors:** Takaaki Morimoto, Masashi Kitagawa, Masaomi Koyanagi, Kenichi Kato, Sadatoshi Tsuzuki, Tetsuro Yamamoto, Keisuke Yamada

**Affiliations:** 1 Department of Neurosurgery, Hyogo Prefecture Amagasaki General Medical Center, Amagasaki, JPN; 2 Department of Dermatology, Amagasaki Yasuda Dermatology Clinic, Amagasaki, JPN; 3 Department of Pathology, Hyogo Prefecture Amagasaki General Medical Center, Amagasaki, JPN

**Keywords:** epidermal inclusion cyst, meningitis, spinal dysraphism, meningocele, cerebrospinal fluid leak

## Abstract

A meningocele is a congenital neural tube defect, and the majority of the meningocele cases are identified perinatally. We present the case of a 67-year-old patient with a sacral meningocele undiagnosed until the removal of a symptomatic epidermal inclusion cyst adjacent to it. Cerebrospinal fluid leakage occurred due to an incision in an undiagnosed meningocele adjacent to the epidermal inclusion cyst. Repair of the cerebrospinal fluid leakage was performed successfully without any deficit. The present case underscores the importance of considering a meningocele as a differential diagnosis for a mass occurring in the midline of the back at any age.

## Introduction

Meningocele is an uncommon neural tube closure defect characterized by the protrusion of a spinal fluid-filled sac lined by meninges through the posterior spina bifida without neural tissue herniation [1]. The majority of cases with a meningocele are identified and treated perinatally, and meningocele is rarely diagnosed in adulthood [2,3].

Epidermal inclusion cysts are harmless, slow-growing bumps under the skin that commonly occur on the face, neck, upper back, scrotum, and genitals although they can occur anywhere. Epidermal inclusion cysts are more frequent in adulthood [4].

Here, we present the case of an elderly patient with a sacral meningocele diagnosed during the removal of a symptomatic epidermal inclusion cyst. Cerebrospinal fluid (CSF) leakage occurred due to an incision in an undiagnosed meningocele adjacent to the epidermal inclusion cyst. Repair of the CSF leakage was performed successfully without any deficit. A meningocele should be considered a differential diagnosis of cysts in the dorsal midline of the body, particularly the buttocks, even in adults.

## Case presentation

**Figure 1:**
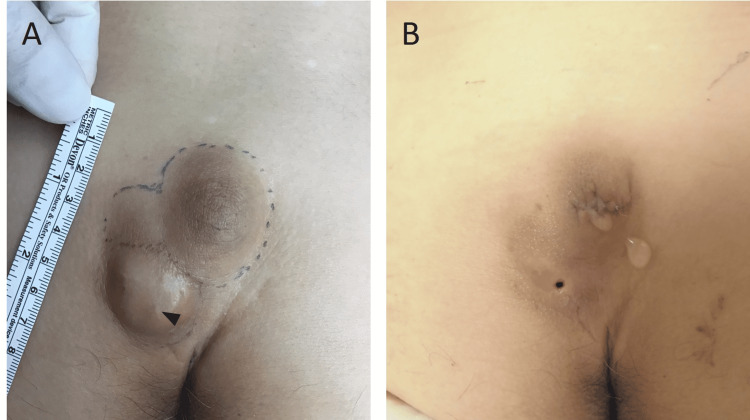
Clinical photograph of the patient. (A) Photograph before the first surgery. A central punctum (arrowhead) was observed in the caudal mass. (B) Photograph after the first surgery. Cerebrospinal fluid leakage was observed only from the cranial incision.

A 67-year-old male without any developmental problems presented with a subcutaneous mass in the buttock (Figure 1A). He had the cyst for nearly five years, and when the mass became painful, he visited a local dermatologist’s office. No neurological symptoms were observed. Ovoid swellings found in the cranial (median, 42 mm) and caudal sides (paramedian, 28 mm) of the gluteal cleft were soft and mobile on palpation. There were no obvious differences on palpation, but upon inspection, the surface of the paramedian caudal swelling was red in color and the central punctum was detected, while the caudal median swelling was slightly dark and the central punctum was not detected. The patient was then diagnosed with an epidermal inclusion cyst. Ultrasound examination revealed two cysts and there was no communication between the two cysts. First, the paramedian caudal cyst was resected. Upon incision, pus drainage was observed initially. Because the quantity of the discharge was not enough, an additional incision on the midline cyst was performed. Subsequently, leakage of transparent liquid was observed.

**Figure 2:**
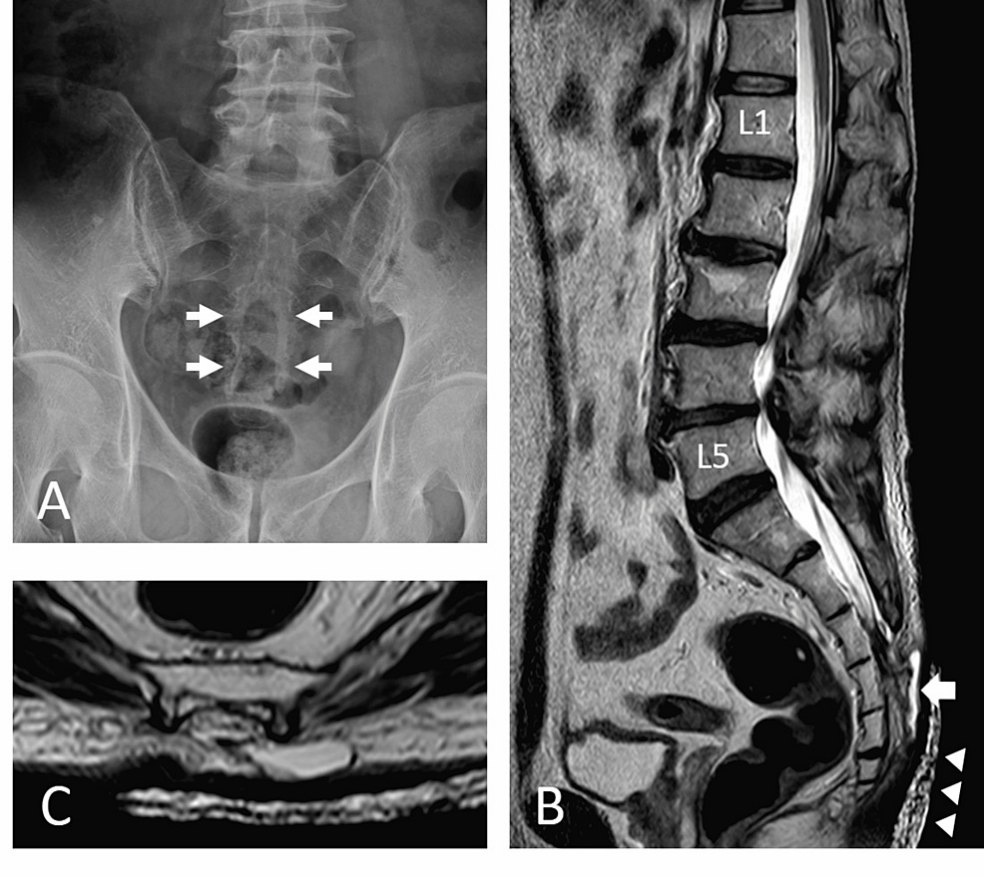
Radiographic image findings. (A) Lumbosacral spine X-ray radiography revealed spina bifida at S3 and below (arrow). (B, C) Magnetic resonance imaging revealed a cyst-like structure (arrow) under the skin at the S3–S5 level. The terminal end of the dural sac and soft tissue contrast were continuous. Tethered cord and lipoma were not detected. Arrowhead: water-absorbing sheet.

**Figure 3:**
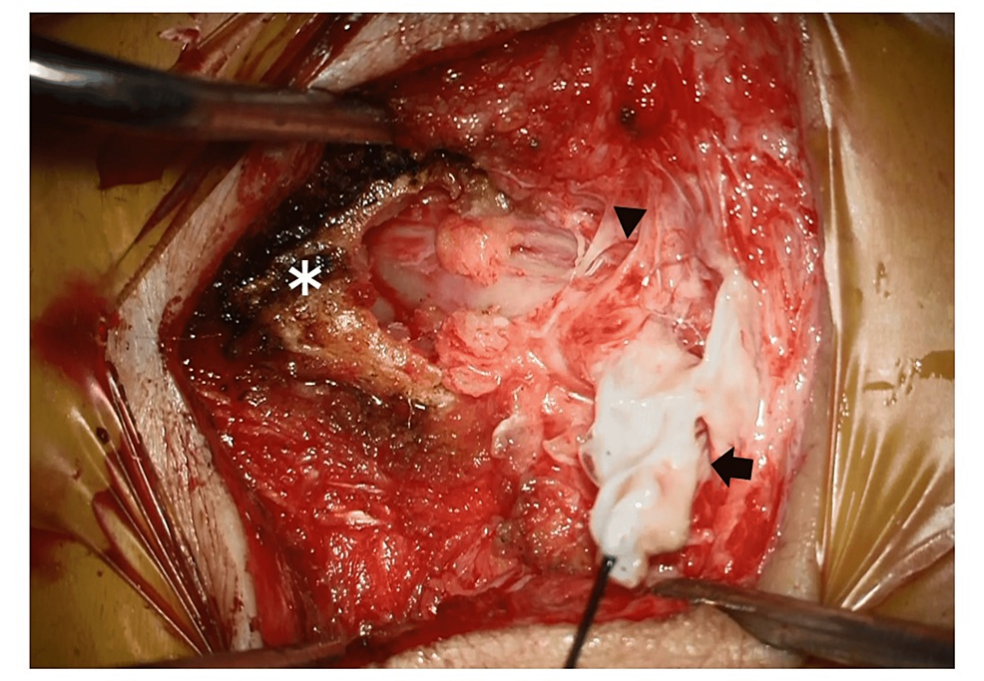
Operative photography. Arrowhead: the lower end of the dural sac. Arrow: meningocele. *: the lower end of the combined sacrum.

As the leakage persisted, the patient was referred to our hospital the day after the surgery. Fluid leakage was observed from only the cranial incision on the midline cyst and not from the caudal cyst (Figure 1B). Lumbosacral spinal X-ray radiography revealed spina bifida of the sacrum (Figure 2A). Magnetic resonance imaging (MRI; Figure 2B, 2C) revealed a cyst-like structure under the skin at the S3-S5 level. The terminal end of the dural sac and soft tissue contrast were continuous, suggesting the collapse of the meningocele. As a result, we diagnosed that the fluid leakage was CSF leakage from the incised meningocele. Emergency surgical repair was performed for CSF leakage. A cyst continuous with the dural sac was identified, which was ligated and dissected (Figure 3).

**Figure 4:**
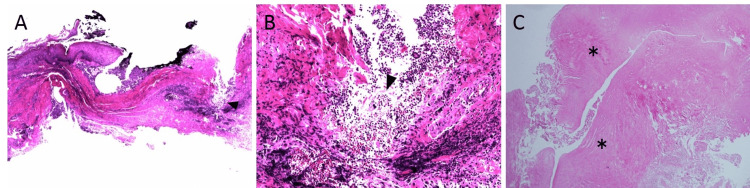
Histopathological findings. (A and B) Photomicrograph of the pathology of the first surgery showing granulation tissue and keratin material (arrowhead). A, hematoxylin-eosin (H-E) staining, ×20 original magnification, B, ×100 original magnification. (C) Photomicrograph of the pathology of the second surgery showing dense fibrous tissue (asterisk). H-E staining, ×40 original magnification.

The histological findings of the paramedian caudal cyst resected at the first surgery by the local dermatologist showed granulation tissue and keratin material, suggesting an epidermal inclusion cyst (Figure 4A, 4B). However, histological findings of the second surgery at our department to repair CSF leakage revealed dense fibrous tissue, which was considered the dura mater. No epithelial component was identified, which suggested a meningocele (Figure 4C). We considered that the epidermal inclusion cyst incidentally occurred adjacent to the sacral meningocele, and when the epidermal inclusion cyst was removed, the meningocele was also incised, resulting in CSF leakage. There was no communication between the two cysts, which was determined based on the absence of CSF leakage upon resection of the epidermal inclusion cyst during the first surgery and even after its incision. Following surgery, CSF leakage disappeared. CSF culture was positive for Enterococcus faecalis, and the patient was treated with vancomycin for two weeks. He was discharged without any deficit.

## Discussion

A meningocele is the simplest form of an open neural tube defect, and it accounts for 2.4% of closed spinal dysraphism cases [5]. The majority of meningoceles are identified and treated perinatally [2,3]. In adults, meningoceles can be detected only when a tethered cord syndrome becomes symptomatic or incidental radiological workups are performed. Moreover, many meningoceles are located in the anterior sacral and dorsal cervical area [3]. To date, only eight adult dorsal lumbosacral meningocele cases, including the present case, have been reported in the literature (Table 1) [2,3,6-8]. Most of the reported cases were of patients in their 20s. To the best of our knowledge, our case is the first case of dorsal meningocele presenting in an elderly person. In the present case, there were no symptoms associated with the meningocele, suggesting that it was not diagnosed until the patient was older.

**Table 1:** Reported cases of an adult lumbosacral meningocele in the English literature

Age at diagnosis/sex	Neurological symptoms	Imaging findings
21/M [6]	Altered sensation of leg, leg length discrepancy	Lumbosacral meningocele, corpus callosal agenesis, conus reaching at L5 on MRI
21/M [6]	Tethered cord syndrome	Lumbosacral meningocele, split cord malformation at L1, conus reaching at L3 on MRI
25/M [3]	Pain, nocturnal enuresis, loss of perianal sensation and ankle jerks	Lumbosacral meningocele, spina bifida at L4 and L5 on X-ray
28/F [7]	Absent ankle reflexes, decreased plantar flexion, no perianal sensation, decreased sensation at S1	Sacral meningocele at S1, conus reaching L5 level, syringomyelia at L2–3 on MRI
48/M [2]	Difficulty in bowel or bladder function, decreased perianal sensation, intact strength in all muscle groups and no sensory abnormality	Lumbosacral meningocele from L5 spina bifida, conus reaching L5 level on MRI
53/M [3]	Low back pain radiating to both lower limbs, urinary incontinence, decreased perianal sensation	Lumbar meningocele, conus reaching at L3 on MRI
53/M [8]	Difficulty in bowel or bladder function, progressive weakness of lower limb	Lumbosacral meningocele, bony defect from L5 to upper sacrum on X-ray
67/M (present case)	Asymptomatic	Meningocele from S3 to S5 spina bifida on MRI

In the present case, an epidermal inclusion cyst existed adjacent to an undiagnosed sacral meningocele. Epidermal inclusion cysts occur anywhere in the body, and the buttock is one of the most common sites. Although a meningocele is extremely rare in the elderly, as described above, the possibility of encountering a meningocele should be considered in differential diagnoses of an epidermal inclusion cyst in the buttock.

Many cases with an epidermal inclusion cyst tend to be treated by an office surgeon or dermatologist. However, the incision of a meningocele will lead to CSF leakage. Because CSF leakage can lead to severe sequelae such as meningitis, subdural hematoma, and cerebral venous thrombosis [9,10], corrective procedures should not be performed in the office clinic.

In many cases, ultrasound scanning is useful to differentiate the meningocele from an epidermal inclusion cyst. Since a meningocele contains CSF, it appears as a uniform low-echoic cyst. In contrast, an epidermal inclusion cyst generally exhibits inner echogenicity, reflecting debris [4], and nearly 96% of these cysts are associated with posterior sound enhancement [11]. However, in real clinical settings as in our case, ultrasound scanning might not be able to differentiate them clearly.

Generally, CT or MRI is required to diagnose an epidermal inclusion cyst with a nonspecific appearance [4]. However, these modalities are often not available in clinics. Another solution could be plain radiography of the lumbosacral area since meningocele accompanies spina bifida. If the cyst is located in the dorsal midline with spina bifida, referral to an appropriate institution for further radiological investigation such as CT or MRI is recommended.

## Conclusions

We encountered a rare case in which an epidermal inclusion cyst occurred adjacent to a sacral meningocele with a delayed presentation in an elderly person. A meningocele can be present at any age, and it should be considered as a differential diagnosis of a cyst in the dorsal midline of the body in both children and adults. In particular, surgical treatment should be performed only if the absence of spinal meningeal cysts involving CSF or neural tissue can be ruled out.
